# Green Synthesis of Composite Graphene Aerogels with Robust Magnetism for Effective Water Remediation

**DOI:** 10.3390/ma12244106

**Published:** 2019-12-08

**Authors:** Qixia Liu, Shiqi Hu, Zhilian Yang, Xueyan Zhang, Jianlong Ge

**Affiliations:** National & Local Joint Engineering Research Center of Technical Fiber Composites for Safety and Health, School of Textile and Clothing, Nantong University, Nantong 226019, China; lqx@ntu.edu.cn (Q.L.); hushiqi477@163.com (S.H.); yzl9723@126.com (Z.Y.); zhangxy@canasin.com (X.Z.)

**Keywords:** magnetic materials, graphene aerogels, chemical reduction, water remediation, photo-Fenton catalysis

## Abstract

Graphene-based three-dimensional (3D) magnetic assemblies have attracted great research attention owing to their multiple natures inherited from 3D graphene assemblies and magnetic materials. However, at present, the practical applications of graphene-based magnetic materials are limited by the relative complex synthesis procedure and harsh operation conditions. Hence, a facile and green synthesis strategy is highly desired. Herein, a magnetic graphene aerogel with magnetite nanoparticles in-situ synthesized on the surface of its frameworks was fabricated through a green and facile strategy. The synthesis process was performed in a gentle condition with low energy consumption. The obtained graphene aerogels exhibited superior magnetism with a saturation magnetization of 55.7 emu·g^−1^. With the merits of well-developed pore structures, high surface area, and robust magnetic property, the obtained composite aerogels exhibited intriguing adsorption and photo-Fenton catalytic degradation performances for the organic dyes in water. Moreover, the utilized graphene aerogels could be recycled from the water due to their effective magnetic separation performance, indicating a promising capability for practical applications in the area of water remediation. We anticipate this synthesis strategy could provide some guidance for the design and development of 3D magnetic assemblies.

## 1. Introduction

In recent years, graphene-based porous materials have attracted tremendous research interests owing to their exceptional properties of huge surface area, large delocalized π-electron system, and excellent mechanical/thermal stability [1]. These promising properties, together with the ease of processability and functionalization, render graphene and graphene-based composite materials to be ideal adsorbents in solid-phase extractions, which is of great importance for water remediation [2,3]. As a result, a great number of functionalized graphene composite materials have been invented aiming to remove the pollutants (e.g., oil, dyes, and heavy metals) from water to deal with the serious environmental problems, such as oil spill and drinking water contamination, which will cause serious threats for the health of human beings [4,5,6,7,8]. Among those materials, graphene aerogels (GAs) with three-dimensional (3D) framework structures exhibit superior performance owing to their merits of excellent media transport property, enhanced adsorption capacity, low density, and convenience to be assembled to devices [9,10]. Consequently, the GAs have played various important roles for water remediation applications, such as the adsorbents, catalysts, and even the electrodes for electrochemical reactions [11,12,13,14].

To meet the increasing requirements for different applications, especially for water remediation, combining graphene with other functional materials, such as polymers [9,15], metals [16,17], and metal oxides [18,19], to synthesize GAs with specific properties has become a facile and effective strategy to improve the service performances of GAs. As a newly developed magnetic material, magnetic composite GAs (M-CGAs) inherit both of the advantages of GAs and magnetic materials; thus, they can be well-regulated by using an external magnetic field, and many specific functions could be realized, such as directed media transporting and magnetic separation [12,20,21]. As a result, the collection and recycling of the adsorbents or catalysts from the reaction systems after utilization become much more efficient and safer. Therefore, the design and fabrication of GAs with magnetic property are very meaningful not only for scientific researches, but also for industrial productions. Up to now, there have been several works reporting the fabrication of GAs with magnetic property. However, most of them were synthesized based on hydrothermal and calcination methods, which are quite complex and highly energy consuming [8,22,23,24]. Therefore, it is urgent to develop a facile and green strategy to synthesize GAs with required magnetic property.

Herein, we report a facile and effective strategy for the fabrication of GAs with intriguing magnetism through a chemical reduction procedure without further calcination or hydrothermal treatment. The key point for this method is the in-situ formation of magnetic metal oxides on the surface of graphene oxides (GO) along with the simultaneous reduction of GO and assembly of GAs. The obtained composite GAs possessed well-developed open-cell structures, and the in-situ synthesized ferromagnetic particles were uniformly and firmly anchored on the surface of graphene. As a result, the obtained GAs exhibited a superior magnetic property. Given the intriguing porous structures, functional ferric oxides, and robust magnetic property, the as-prepared M-CGAs were further employed for the water remediation application, and the results showed that the M-CGAs were capable of removing the organic dyes from water. Besides, a plausible mechanism of adsorption and photo-Fenton catalytic degradation for organic dyes was proposed to describe the organic removal process. We anticipate that this strategy could offer some guidance for the development of composite GAs with designed functionalities for different applications.

## 2. Materials and Methods

### 2.1. Materials and Chemicals

The graphite powder and KMnO_4_ were purchased from Jiangsu Yonghua Chemical Technology Co., Ltd. The 98 wt.% H_2_SO_4_, H_2_O_2_ (30%), and FeCl_2_·4H_2_O were provided by Shanghai Lingfeng Chemical Reagent Co., Ltd. FeCl_3_·6H_2_O was purchased from Sinopharm Group Chemical Reagent Co., Ltd. NH_3_·H_2_O was provided by Jiangsu Yangzhou Hubao Chemical Reagent Co., Ltd. NaHSO_3_, rhodamine B (RhB), methylene blue (MB), and methyl orange (MO) were provided by Shanghai Runjie Chemical Reagent Co., Ltd. Ultra-pure water was obtained from a water purification system. All the chemicals were used without further purification [25].

### 2.2. Synthesis Methods

#### 2.2.1. Synthesis of GO

GO was prepared by using a modified Hummers’ method [25]. In a typical procedure, 2 g graphite powder was dispersed in 46 mL of H_2_SO_4_ (98 wt.%) in an ice bath under vigorous stirring. After that, 6 g KMnO_4_ powder was slowly added into the mixture for several times, and the temperature of the reaction system was strictly controlled to be <5 °C. After adding the KMnO_4_ powder, the mixture was put into a water bath with a temperature of 40 °C for oscillation. After 30 min, the dark green mixture was heated at 95 °C for 15 min. After that, 300 mL deionized water and 10 mL 30% H_2_O_2_ were slowly added into the reaction system, respectively, to terminate the reaction. At last, 300 mL of mixture of hydrochloric acid (36 wt.%) and deionized water with a mass ratio of 1:9 was added. After 30 min ultrasonic treatment, the mixture was washed to neutral by using deionized water. Finally, the obtained GO slurry was dried at 60 °C.

#### 2.2.2. Synthesis of M-CGAs

**Figure 1:**
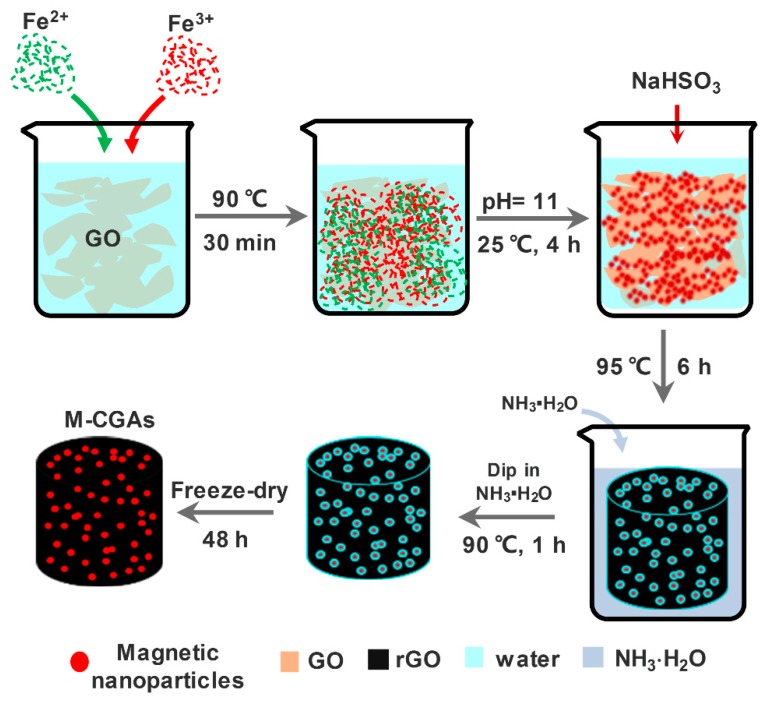
Schematic illustration demonstrating the synthesis process of magnetic composite graphene aerogels (M-CGAs).

In a typical procedure (Figure 1), 0.11 g FeCl_3_·6H_2_O and 0.18 g FeCl_2_·4H_2_O were dissolved in 20 mL deionized water to obtain solution A. Then, 0.1 g GO was dispersed in 200 mL deionized water under ultrasonic treatment (350 W) for 2 h to obtain solution B. After that, the as-prepared solution A was added into solution B within 5 min by drops, and the obtained mixture was stirred for 30 min at 90 °C by use of a water bath. After cooling to room temperature, the pH value of the mixture was adjusted to 11 by using the ammonia solution and stirring continued for 4 h. After that, the mixture was collected with the assistance of a magnet; the supernatant liquid was almost colorless (Figure S1). The obtained composite materials of GO and iron species were washed by distilled water and anhydrous ethanol for three times and then dried at 200 °C. After that, a certain amount of the obtained magnetic composite was mixed with deionized water to obtain a solution with solid content of 2 mg mL^−1^ and ultrasonic treated for 15 min. For the in-situ reduction of GO, 120 mg NaHSO_3_ was added into 20 mL of the obtained solution, and put under ultrasonic treatment for 15 min with the power of 300 W. After that, a gelatinous graphene composite was obtained by heating the solution in a water bath with temperature of 95 °C for 6 h. After being washed by distilled water for several times, the gelatinous composite was impregnated in 14 wt.% ammonia solution for 1 h at 90 °C, and then the composite was freeze-dried at −80 °C for 48 h to obtain the M-CGAs. Meanwhile, pristine GAs without loading iron species were also prepared through the same procedure in order to compare studies.

### 2.3. Characterization

The morphologies and components of the as-prepared materials were systematically characterized by using various measurement methods. A field-emission scanning electron microscope (FE-SEM, Hitachi S-4800, Japan) equipped with energy-dispersive spectroscopy (EDS, INCA ENERGY 350, Oxford, UK) was employed to characterize the morphologies and surface elements distribution of the obtained materials with an acceleration voltage of 15 kV. The particle size and micro-crystallized structures of the synthesized composites were characterized by using a high-resolution transmission electron microscope (TEM, JEM-2100F, JEM, Japan) with the acceleration voltage of 200 kV. X-ray diffraction (XRD, Ultima IV, Rigaku, Japan) with a Cu Kα radiation source generated at 40 kV and 40 mA was used to verify the crystal structure and phase compositions of the obtained materials. Fourier transform infrared spectroscopy (FT-IR, Nicolet iS 50, Thermofisher Scientific, USA) was used to obtain the FT-IR spectra. Inductively coupled plasma massspectrometry (ICP-MS, NexION 350, PerkinElmer, USA) was used to analysis the content of Fe ion in solutions. The surface chemical compositions were examined by employing X-ray photoelectron spectroscopy (XPS, PHI-5702, Physical Electronics, USA). Al Kα was used as the excitation source; the spot size was 400 μm, the pass energy was 30.0 eV, and the energy step size was 0.05 eV. The magnetic property of the as-prepared samples was evaluated at room temperature by using a vibrating sample magnetometer (VSM, 7307, Lake Shore, USA). The surface area and pore structures were characterized by using a surface area and pore analyzer (ASAP2020, Micromeritics, USA) based on the N_2_ adsorption–desorption isotherms and, before testing the samples were degassed at 200 °C for 5 h to remove the adsorbed H_2_O.

### 2.4. Water Remediation Performance Evaluation

#### 2.4.1. Adsorption Behavior

The adsorption behavior of the as-prepared samples for pollutants in water were estimated by using RhB as the model at room temperature. In general, the as-prepared samples were directly put into the RhB aqueous solutions with certain concentrations for a designed time. The dosage of sample was fixed to 0.2 g L^−1^. The pH of solutions was adjusted to 5 to obtain an optimized adsorption condition. The residual RhB content of the aqueous solutions was estimated by using a UV–Vis spectrophotometer (TU-1900, PERSEE, China). The adsorption efficiencies were calculated by the following Equations:

(1) $$q_{t}=\frac{\left(C_{0}-C_{t}\right)\times \;V}{m}$$

(2) $$q_{e}=\frac{\left(C_{0}-C_{e}\right)\times V}{m}$$

(3) $$R=\frac{\left(C_{0}-C_{e}\right)}{C_{0}}\times 100\%$$

where $q_{t}$ is the adoption capacity at the time *t*, $C_{0}$ is the initial concentration of dyes aqueous solutions, $C_{t}$ is the concentration of dyes aqueous solutions at the time *t*, $q_{e}$ is the equilibrium absorption capacity, $C_{e}$ is the concentration of dyes aqueous solutions when the adsorption reached equilibrium, ***R*** is the removal ratio, and *m* is the weight of the used samples.

#### 2.4.2. Photo-Fenton Catalytic Activity

To evaluate the photo-Fenton catalytic performance of the as-prepared samples, a setting amount of H_2_O_2_ (10 mmol L^−1^) was added into the dye solutions (pH = 7) with the selected catalysts. The dosage of M-CGAs was 0.2 g L^−1^. The whole reactions were performed under the irradiation of a 300 W Xe lamp. The distance between the lamp and solution was fixed at ~15 cm. During the reaction time, a water circulation refrigeration system was used to set the test temperature at 25 ± 3 °C. The content of residual dyes in water was examined by using the UV–Vis spectrophotometer.

#### 2.4.3. Regeneration of M-CGAs

The regeneration of used M-CGAs was carried out through a solvent desorption method with ethanol. After each testing cycle, the used adsorbent was separated from water using a magnet and put into ethanol for 3 h and stirred. After completely drying in a vacuum oven, the regenerated M-CGAs were used for the next cycling test.

## 3. Results and Discussion

**Figure 2:**
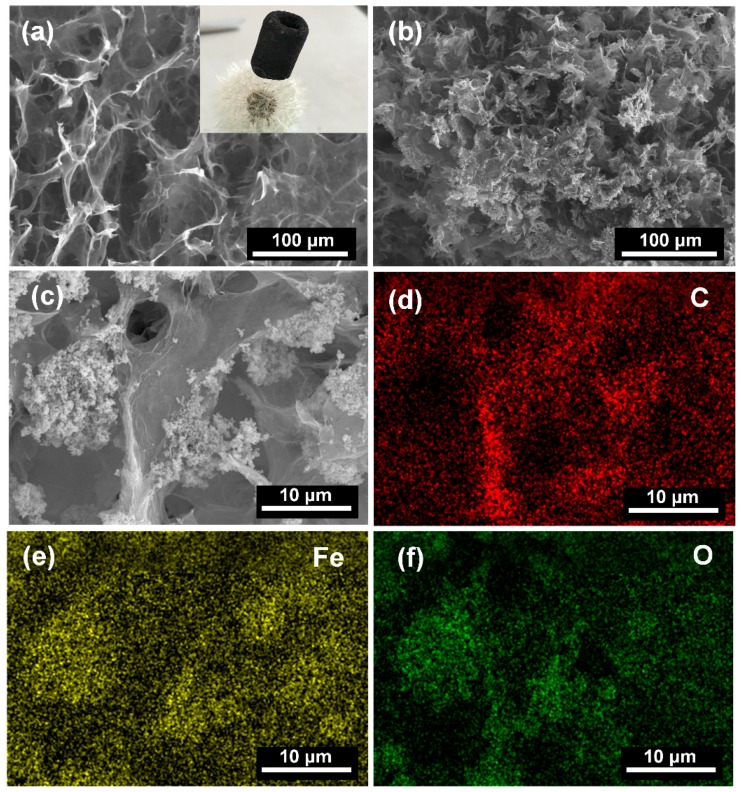
(**a**) The SEM image of the as-prepared pristine graphene aerogels (GAs) (inset is the optical photograph of the pristine graphene aerogel). (**b**,**c**) SEM images of the as-prepared M-CGAs. (**d**–**f**) Elemental mapping of the selected area of the M-CGAs.

The micro morphologies of the as-prepared pristine GAs and M-CGAs were characterized by SEM analyses. The SEM images shown in Figure 2a indicate that the as-synthesized pristine GAs possessed well-developed open-cell structures with intact interconnected frameworks. The honeycomb-like open-cell structures could be observed through the SEM. Meanwhile, the optical photograph of the GA inset in Figure 2a demonstrates that the as-prepared GAs can be easily held up by the tomentum of dandelion seeds, indicating an ultralight property of GAs, which was attributed to the small solid content and low density of graphene [9]. In general, the low density of an adsorbent could ensure a high weight-based adsorption capacity [26]. Figure 2b shows the open-cell structures of the as-prepared M-CGAs. It can be found that the frameworks remain intact and stable with the loading of numerous nanoparticles; the slightly decreased size of open-cells may be attributed to the blockage of the particles. Figure 2c shows that the nanoparticles are firmly anchored on the surface of graphene; meanwhile the higher magnified SEM image (Figure S2) demonstrates that the particles were formed by numerous nanocrystals. Elemental mapping images shown in Figure 2d–f indicate that the elemental composition of the M-CGAs are C, Fe, and O, while the particles are mainly consisting of Fe, and O.

**Figure 3:**
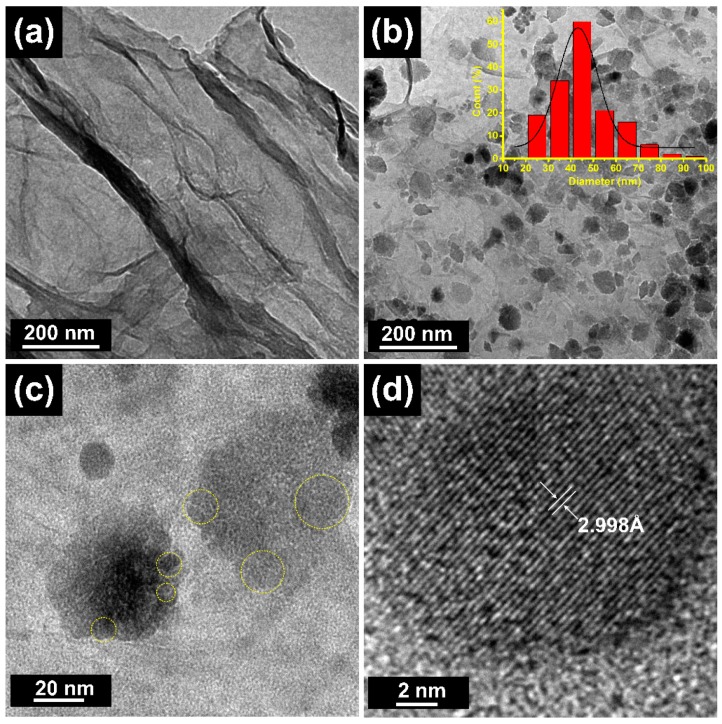
TEM images of (**a**) pristine GAs. (**b**) M-CGAs (inset is the size distribution curve of the nanoparticles). (**c**,**d**) HRTEM image of M-CGAs.

To further verify the microstructures and compositions of GAs and M-CGAs, TEM characterizations were conducted. As shown in Figure 3a, the TEM image of pristine GAs depicts a well-exfoliated graphene layer without any other composites on its surface; while, for M-CGAs, numerous nanoparticles can be found on the surface of frameworks, which is consistent with the above-mentioned SEM results. As depicted by Figure 3b, the size distribution of those particles is in the range of 20–100 nm, and the average diameter was ~45 nm, indicating a good uniformity. Interestingly, the high resolution TEM (HRTEM) images shown in Figure 3c demonstrate that the particles consist of various nanocrystals with smaller size. This phenomenon is consistent with the results of the SEM images. Figure 3d depicts that those nanoparticles exhibit well crystalline textures, with a representative lattice spacing of 2.998 Å, which may be ascribed to the (220) plane of Fe_3_O_4_ crystals [27].

**Figure 4:**
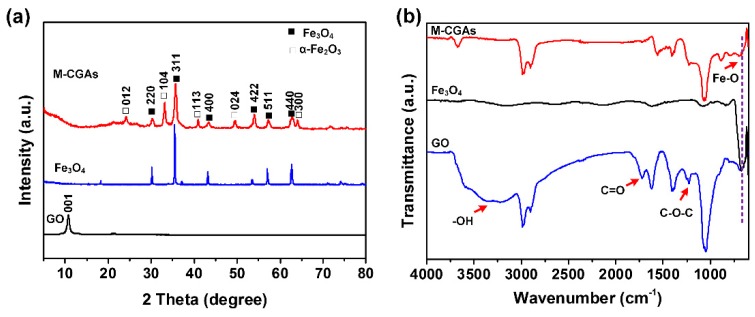
(**a**) XRD and (**b**) FT-IR spectra of GO, Fe_3_O_4_, and M-CGAs.

To further reveal the compositions, the XRD patterns of the as-prepared GO and M-CGAs were obtained. As shown in Figure 4a, it can be found that the XRD pattern of GO exhibits a typical diffraction peak at 10.6°, which is corresponded to the (001) plane of GO [28]. While for the pattern of M-CGAs, strong diffraction peaks at 30.4°, 35.6°, 43.4°, 53.9°, 57.3°, and 62.7° emerged, which are ascribed to the (220), (311), (400), (422), (511), and (440) planes of Fe_3_O_4_ crystal structure (JCPDS card no. 19-0629), indicating the successful synthesis of Fe_3_O_4_ [29]. The weak peaks at 24.2°, 33.1°, 40.9°, 49.6°, and 64.1° may be corresponded to the (012), (104), (113), (024), and (300) planes of α-Fe_2_O_3_ (JCPDS card no. 33-0664), which is widely used for catalytic reactions [30,31]. FT-IR spectra were also used to characterize the evolution of surface functional groups of the obtained materials. As can be recognized from Figure 4b, in the spectrum of GO, the strong and wide peak shift at ~3223 cm^−1^ is ascribed to the –OH stretching vibration absorption, and the stretching vibration peaks of C=O and C-O-C appeared at 1721 cm^−1^ and 1226 cm^−1^, respectively. As expected, the above-mentioned stretching vibration peaks of oxygen-containing functional groups disappeared in the spectrum of M-CGAs, indicating that the GO had been well-reduced. Moreover, the stretching vibration peak at 672 cm^−1^ is assigned to Fe-O [31], which is consistent with the result of XRD.

**Figure 5:**
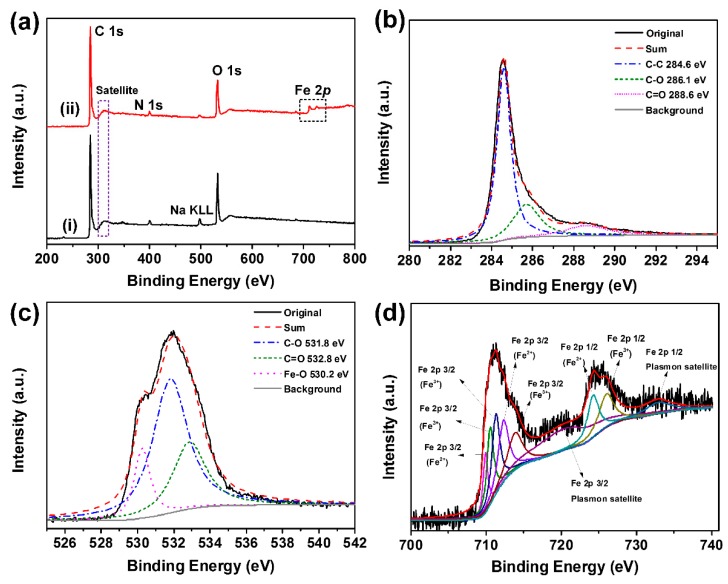
(**a**) XPS full survey scan spectra of the GAs and M-CGAs. (**b**–**d**) C1s, (**c**) O1s, (**d**) Fe2p spectra of M-CGAs.

The fine chemical structures and elemental compositions of the obtained GAs and M-CGAs were further analyzed by XPS. As shown in Figure 5a, in the survey spectra of pristine GAs and M-CGAs, the prominent peaks at 285 and 310 eV correspond to the C1s and its plasmon satellite. The peak at 400 eV is assigned to N1s, which was derived from the treatment of NH_3_·H_2_O. The peak around at 532 eV corresponds to O1s; meanwhile, for the M-CGAs, peaks at 712 and 725 eV could be observed, which are attributed to the existence of iron oxides [32]. Further analysis on the prominent peaks in the spectra of M-CGAs was performed. As shown in Figure 5b, the C1_S_ peak region could be assigned to the C-C (284.6 eV), C-O (286.1 eV), and C=O (288.6 eV). Figure 5c demonstrates the O1s peak region could be ascribed to C-O (531.8 eV), C=O (532.8 eV), and Fe-O (530.2 eV), which is consistent with the results of FT-IR analysis. In Figure 5d, the peak at 712 eV could be assigned to Fe^2+^2$p_{3/2}$ and Fe^3+^2$p_{3/2}$; meanwhile, the peak at 725 eV may be attributed to the Fe^2+^2$p_{1/2}$ and Fe^3+^2$p_{1/2}$ [33], indicating compounds of various ferric oxides, and the proportion of amorphous Fe^3+^, Fe_3_O_4_ (FeO·Fe_2_O_3_), and Fe_2_O_3_ are calculated as 42.75 atomic%, 33.45 atomic%, and 23.8 atomic%, respectively. The above-mentioned XPS result is in accordance to the analysis of XRD.

**Figure 6:**
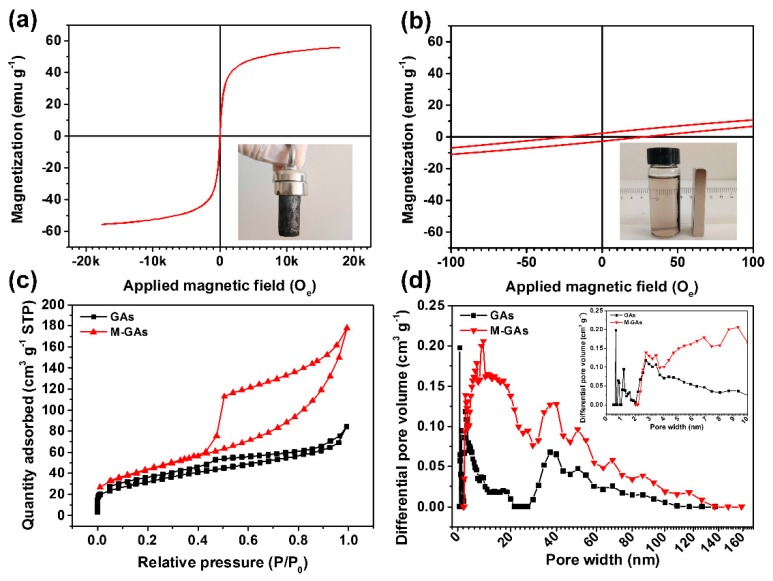
(**a**,**b**) Magnetic hysteresis loops of M-CGAs (inset is the optical photograph demonstrating the robust magnetic property of the M-CGAs). (**c**) Nitrogen adsorption–desorption isotherms. (**d**) Pore size distribution curves of the pristine GAs and M-CGAs, respectively.

With the presence of magnetic nanoparticles, the as-prepared M-CGAs exhibited an expected magnetic property. As shown in Figure 6a,b, the obtained M-CGAs exhibited an intriguing magnetic response behavior, and the corresponded saturation magnetization is as high as 55.7 emu·g^−1^, which is superior to those of the reported works [23,32,34,35,36]. Moreover, the remaining saturation magnetization and coercive forces were just 2.4 emu g^−1^ and 24 O_e_, respectively, which are the typical properties of a soft magnetic material. The robust magnetic property endows M-CGAs an intriguing magnetic separation performance in liquid. As shown in Figure 6b, the inset optical photograph depicts that the M-CGAs in water could be quickly separated by the magnet even at a distance of 1 cm away, indicating a good magnetic separation property.

The surface area and pore structures of the obtained materials were carefully studied through N_2_ adsorption at 77 K to further evaluate the possibility of M-CGAs as a water remediation material. Figure 6c depicts the nitrogen adsorption–desorption behavior of pristine GAs and M-CGAs. As can be seen, the nitrogen adsorption–desorption isotherms of pristine GAs and M-CGAs exhibit similar shape, which combine the characteristics of type I and type IV according to the IUPAC classification, in which the adsorption–desorption behaviors including micropore filling, monolayer adsorption, multilayer adsorption, capillary condensation, as well as obvious H4 hysteresis loops could be observed. The rapid increment of N_2_ adsorption amount at the region of low pressure (<0.1 P/P_0_) may be attributed to the existence of microspores causing the micropore filling behavior. The continuous increased N_2_ adsorption amount, as well as the hysteresis loops in the region of 0.1 < P/P_0_ < 0.9, indicate the formation of mesopores, while the obvious uptake of N_2_ adsorption amount and hysteresis loops occurring at P/P_0_ > 0.9 reveals that most of the pores are in the shape of opened-slit, which are beneficial for the transport of media [37]. Moreover, it can be seen that both the N_2_ adsorption amount and the area of hysteresis loop of M-CGAs are obviously increased compared with those of pristine GAs. This phenomenon may be contributed by the increased amount of mesopores.

In order to further investigate the pore structures of the as-prepared materials, a nonlocal density functional theory (NLDFT) method was employed to quantitatively analyze the pore size distribution (PSD) and pore volume based on the obtained N_2_ adsorption–desorption isotherms. As can been seen from Figure 6d, the PSD curve of GAs demonstrate that the pore sizes of GAs are mainly in the ranges of 0–20 nm and 30–120 nm. In addition, there are polydisperse peaks in both <2 nm (micropores) and 2–50 nm (mesopores), confirming the existence of both micropores, mesopores, and macropores. As calculated, the total pore volume of pristine GAs was 0.13 cm^3^ g^−1^, and the proportion of each kind of pores was about 22%, 70%, and 8%, respectively. As for the M-CGAs, similar phenomenon can be observed from the PSD of M-CGAs, but a strong and wide peak at ~9 nm and the total volume increased obviously. The total pore volume of M-CGAs was 0.22 cm^3^ g^−1^, the BET surface area was about 157 m^2^ g^−1^, and the corresponded proportion of each kind of pore was about 10%, 83%, and 7%, respectively. By comparison, it can be found that the total pore volume and mesoporosity of M-CGAs were significantly increased compared with those of pristine GAs, while the macroporisity and microporosity were dropped. This phenomenon may be caused by the following reasons: (i) the nano-scaled particles generated in-situ on the GO sheet layer blocked some of the micropores; (ii) a large number of slit-like mesopores had generated due to the accumulation of the nanoparticles.

**Figure 7:**
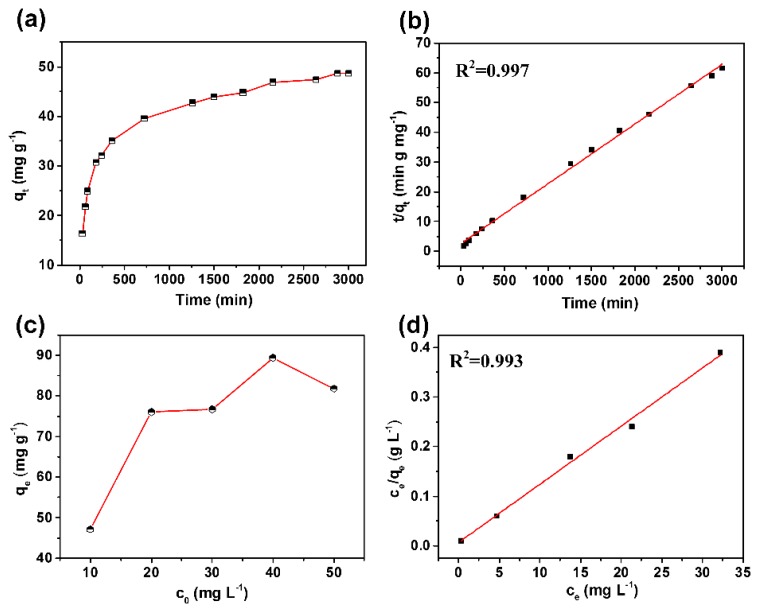
(**a**) Adsorption kinetic curve. (**b**) The fitted curve based on the pseudo second-order rate equation. (**c**) The adsorption isotherm. (**d**) The fitted adsorption isotherm based on Langmuir model.

The facile magnetic separation performance and intriguing pore structures make M-CGAs a promising candidate for water remediation, which encourage us to investigate its adsorption performance for organic molecules in water. In this study, the RhB was selected as a model to test the adsorption performance of the obtained M-CGAs. Figure 7a depicts the corresponded absorption kinetic curve. It can be found that the adsorption rate was really fast in the first 500 min, then the adsorption rate became slow. Upon reaching the equilibrium, the corresponding removal ratio of RhB was 97.4%, and the whole adsorption performance was superior to that of GAs (Figure S3), which could be attributed to the increased surface area and enhanced mesoporous structures providing more active sites for the adsorption of organic molecules. The adsorption kinetics process could be well-described by the pseudo second-order rate equation (Figure 7b), which means that the adsorption rate was mainly controlled by the chemical adsorption process, resulting in a relative long adsorption equilibrium time [38]. Figure 7c shows the adsorption isotherm, from which it can be found that the adsorption capacity increased obviously with the increment of dye concentration, while a decrement was observed when a critical concentration was reached. The highest adsorption of 89.4 mg g^−1^ could be obtained. This adsorption behavior could be well-fitted by the Langmuir adsorption equation (Figure 7d), which means that the dye molecules were monolayer adsorbed [39]. Additionally, the cycled adsorption performance was further evaluated to demonstrate the stability of M-CGAs. The adsorption time of each cycle was 3 h, and then the M-CGAs were separated with the assistance of a magnet and then regenerated. As shown in Figure S4, the RhB removal ratio was relatively stable, and the decrement may be attributed to the loss of adsorbents during the cycling test process. Meanwhile, as shown in Figure S5, the SEM image of the used M-CGAs demonstrates that the magnetic nanoparticles and structures were instantly maintained, and the iron compound detected in the eluate was less than 0.5 mg L^−1^, indicating a good stability of the M-CGAs.

**Figure 8:**
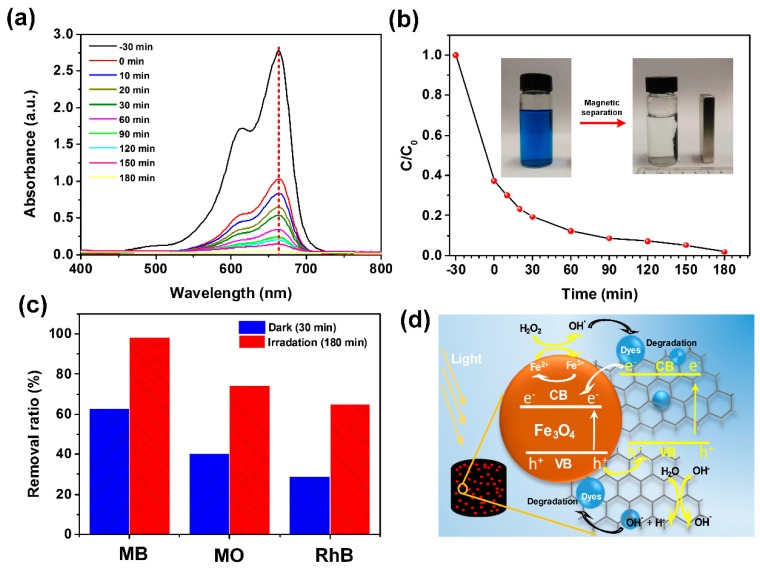
(**a**) The UV–Vis absorption spectra of methylene blue (MB) solutions with M-CGAs under the irradiation of visible-light at different time intervals. (**b**) Removal curve of MB. Inset depicts the robust magnetic separation performance. (**c**) Removal ratio of different organic dyes at the same operation condition. (**d**) Schematic illustration indicating a plausible mechanism of the organic pollutants removed from water by M-CGAs.

Considering the catalytic property of iron oxides [40], the photo-Fenton catalytic degradation performance of M-CGAs over organic dyes in water was also evaluated. Figure 8a is the UV–Vis spectra of the MB solutions with M-CGAs both as the adsorbents and catalysts under different visible-light irradiation time. It can be found that the adsorption intensity at wavelength of 664 cm^−1^ decreased obviously at the first 30 min without light irradiation, which was mainly contributed by the adsorption behavior [41]. As mentioned above, the adsorption rate is mainly controlled by the chemical adsorption process, which is quite slow. We employed visible-light and H_2_O_2_ to induce a typical photo-Fenton catalytic reaction to accelerate the removal of dyes from water. As expected, the dye removal ratio for MB in water could reach more than 98% within 180 min (Figure 8b). Similar effect could also be adapted to MO and RhB in water (Figure 8c). Moreover, as shown by the inset of Figure 8b, the utilized M-CGAs could be facilely separated for recycling by using a small magnet, which is critical for practical applications. In addition, considering the chemical compositions and porous structures of M-CGAs, a plausible mechanism was proposed to explain the removal process of dyes with the presence of M-CGAs. As shown in Figure 8d, at the first stage, the dye molecules were adsorbed based on two kind of interaction forces [40]: (i) the interaction between graphene and dye through electrostatic attraction, π-π stacking, and hydrogen bonding; (ii) the chemisorption of dyes with iron oxides. Meanwhile, the Fenton reactions of iron oxides occurred with the presence of H_2_O_2_ and irradiation of visible-light. As demonstrated by Equations (4) and (6), numerous hydroxyl radicals (OH^▪^) with high oxidizability were generated, therefore the adsorbed organic dyes could be effectively degraded to CO_2_ and H_2_O. Herein, it should be mentioned that the heterojunction structures of iron oxides and graphene could effectively enhance the separation of photo-generated electron-hole pairs due to the potential difference of conduction bands (CB) and valence bands (VB) [40,41,42,43].

(4) $${\mathrm{Fe}}^{2+}+H_{2}O_{2}\to {\mathrm{Fe}}^{3+}+\mathrm{OH}\cdot +{\mathrm{OH}}^{-}$$

(5) $${\mathrm{Fe}}^{3+}+H_{2}O_{2}\to {\mathrm{Fe}}^{2+}+\mathrm{OOH}\cdot +H^{+}$$

(6) $$\mathrm{organics}+\mathrm{OH}\cdot \to {\mathrm{CO}}_{2}+H_{2}O$$

## 4. Conclusions

In this work, we reported a facile and green method to fabricate magnetic 3D graphene assemblies. Magnetite nanoparticles were synthesized in-situ on the surface of GO; meanwhile, the GO sheets were simultaneously assembled and reduced in a gentle condition with low energy consumption. As expected, the obtained M-CGAs possessed a high saturation magnetization of 55.7 emu g^−1^, as well as a small remaining saturation magnetization and a tiny coercive force. With the aid of chemically activated nanoparticles, high surface area, and open-cell structures, the M-CGAs exhibited a typical chemical adsorption behavior with the highest equilibrium absorption capacity of 89.4 mg g^−1^ for organic dye in water. Moreover, the photo-Fenton catalytic activity of the M-CGAs was further employed to enhance its organic removal capability. Upon the irradiation of visible-light, almost 98% of the MB in water could be removed within 180 min, and a plausible mechanism explaining the removal process of dyes from water was proposed. After each process of the adsorption and catalytic degradation, the used M-CGAs could be quickly collected and reused by simply using a small magnet, indicating robust recyclability for practical use.

## Supplementary Materials

The following are available online at https://www.mdpi.com/1996-1944/12/24/4106/s1: Figure S1: Digital photographs demonstrating the co-precipitation system of Fe^3+^/Fe^2+^/GO; Figure S2: High magnified SEM image of the nanoparticles on the surface of graphene; Figure S3: Removal ratio of GAs and M-CGAs for RhB in water at room temperature; Figure S4: RhB removal ratio of M-CGAs and Fe ion content in the eluents of different adsorption-desorption cycles; Figure S5: SEM image of M-CGAs after cycled adsorption-desorption.
