# Supplementary material for: Green Synthesis of Composite Graphene Aerogels with Robust Magnetism for Effective Water Remediation
Source: Materials (Basel). 2019 Dec 8;12(24):4106. doi: 10.3390/ma12244106 (PMC6947391; doi:10.3390/ma12244106)
Supplement: Supplementary file 1 [file materials-12-04106-s001.pdf]

materials

Supplementary Information

# Green Synthesis of Composite Graphene Aerogels with Robust Magnetism for Effective Water Remediation

**Qixia Liu, Shiqi Hu, Zhilian Yang, Xueyan Zhang and Jianlong Ge***

National & Local Joint Engineering Research Center of Technical Fiber Composites for Safety and Health, School of Textile and Clothing, Nantong University, Nantong 226019, China; lqx@ntu.edu.cn (Q.L.); hushiqi477@163.com (S.H.); yzl9723@126.com (Z.Y.); zhangxy@canasin.com (X.Z.)

* Correspondence: gejianlong@ntu.edu.cn; Tel.: +86-513-8501-2836

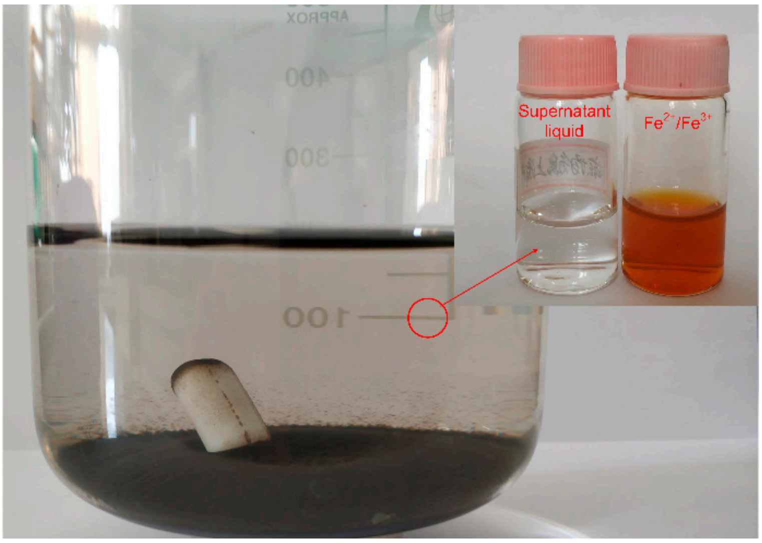


**Figure S1.** Digital photographs demonstrating the co-precipitation system of $Fe^{3+}/Fe^{2+}$/GO. (Inset is the comparison of supernatant liquid and pristine $Fe^{3+}/Fe^{2+}$ solution).

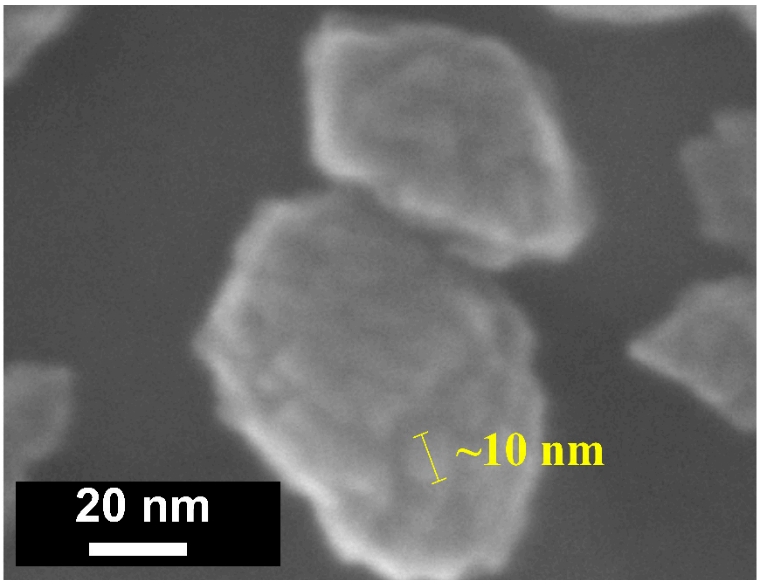


**Figure S2.** High magnified SEM image of the nanoparticles on the surface of graphene.

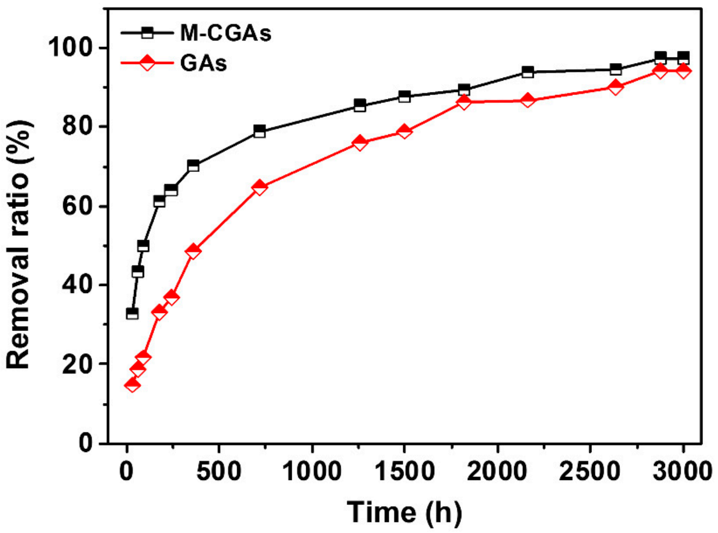


**Figure S3.** Removal ratio of GAs and M-CGAs for RhB in water at room temperature.

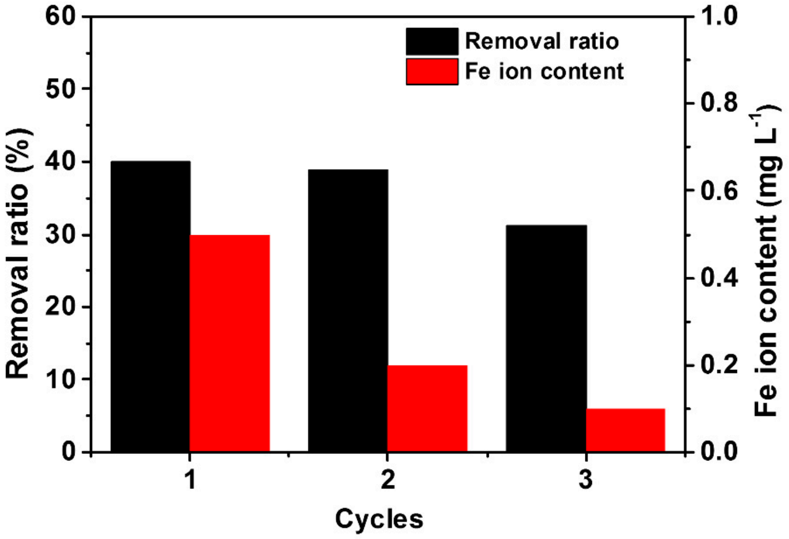


**Figure S4.** RhB removal ratio of M-CGAs and Fe ion content in the eluents of different adsorption-desorption cycles.

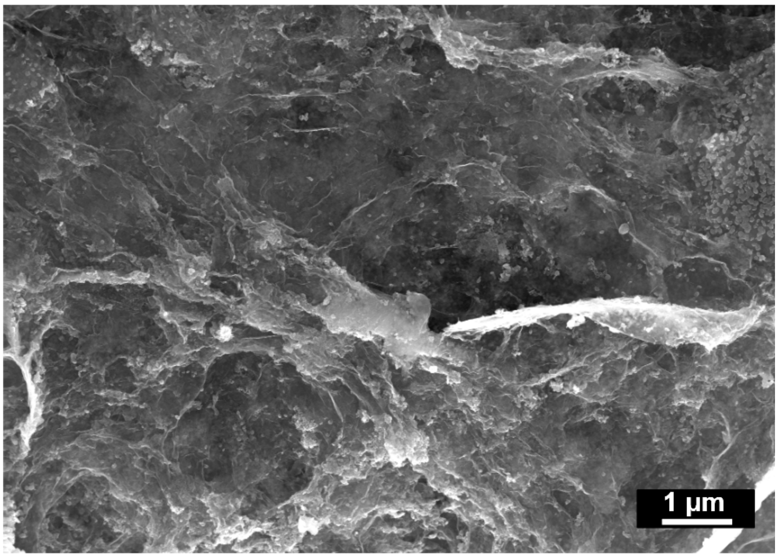


**Figure S5.** SEM image of M-CGAs after cycled adsorption-desorption.
